# Detection of deceptive motions in rugby from visual motion cues

**DOI:** 10.1371/journal.pone.0220878

**Published:** 2019-09-13

**Authors:** Sean Dean Lynch, Anne-Hélène Olivier, Benoit Bideau, Richard Kulpa

**Affiliations:** 1 Univ Rennes, M2S - EA 7470, F-35000 Rennes, France; 2 Univ Rennes, CNRS, Inria, IRISA - UMR 6074, F-35000 Rennes, France; Manchester Metropolitan University - Cheshire Campus, UNITED KINGDOM

## Abstract

Frequently, in rugby, players incorporate deceptive motions (e.g., a side-step) in order to pass their opponent. Previous works showed that expert defenders are more efficient in detecting deceptive motions. Performance was shown to be correlated with the evolution of the center of gravity of the attacker, suggesting that experts may rely on global motion cues. This study aims at investigating whether a representation of center of gravity can be useful for training purposes, by using this representation alone or by combining it with the local motion cues given by body parts. We designed an experiment in virtual reality to control the motion cues available to the defenders. Sixteen healthy participants (seven experts and nine novices) acted as defenders while a virtual attacker approached. Participants completed two separate tasks. The first was a time occlusion perception task, occlusion after 100ms, 200ms or 300ms after the initial change in direction, thereafter participants indicated the passing direction of the attacker. The second was a perception-action task, participants were instructed to intercept the oncoming attacker by displacing medio-laterally. The attacker performed either a non-deceptive motion, directly toward the final passing direction or a deceptive motion, initially toward a false direction before quickly reorienting to the true direction. There was a main effect of expertise, appearance, cut off times and motion on correct responses during both tasks. There was an interaction between visual appearance and expertise, and between motion type and expertise during the perception task, however, this interaction was not present during the perception-action task. We observed that experts maintained superiority in the perception of deceptive motion; however when the visual appearance is reduced to global motion alone the difference between novices and experts is reduced. We further explore the interactions and discuss the effects observed for the visual appearance and expertise.

## Introduction

Rugby, as per field-based team sports, is characterized by brief high-intensity efforts of running and acceleration over longer low-intensity periods [1]. The game consists of two teams competing over two 40-minute halves for the highest accumulation of points scored, with the greatest number of points being awarded for carrying the ball over the opponents try-line. Therefore, a primary objective of the game is to gain territory by advancing the ball towards the opponents try-line. In order to prevent the advancement of the attacking team (the ball carrying team) and possibly gain possession, the defending team will tackle the player that is carrying the ball. A tackle as defined by the international governing body of rugby is when the ball-carrier is held by one or more opponents and brought to the ground. Due to this nature, a proportion of the game consists of tackling [2, 3], where an increased number of tackles has been associated to game success rate [4, 5]. To gain an attacking advantage, attackers can use their bodily movements to generate deceptive motion (e.g., a side-step in rugby), misleading the defender with the intention to run in one direction while actually intending to run in the other direction [6–10]. While generally, motor control and orientation of the body is coordinated according to a top-down strategy of head and gaze alignment before body reorientation [11], in a sport-specific context of deceptive motion Brault *et al*. [7] reported a bottom-up strategy, where a displacement of support is prior to upper trunk and head reorientation. Further, Brault *et al*. [7] reported that the center of mass displacement on the medio-lateral plane was minimized, it was the upper limbs, head, and trunk that were organized for deception. Although trunk yaw was similar between deceptive and non-deceptive motion, the upper trunk angular movement changes (i.e., shoulders) would be exaggerated in deception.

Within the attacker-defender dyad, while the attacker intends to deceive the defender with specific body motion, the defender utilizes the relevant visual cue information available to intercept the attacker [12]. Previous studies demonstrated the ability of observers to distinguish between a deceptive and non-deceptive motion of another person [13]. Using point-light displays, participants were required to observe actors lifting boxes of various weights. It was reported that the participants could perceive the action of a box being picked up, even from a limited point-light display, further, also the relative weight and if the actor was attempting to deceive the observer about the true weight.

In a sports specific context, authors showed that the ability to perceive and to correctly anticipate an opponent motion is related to the level of expertise (see [6], [14], [15], and [10]). Jackson *et al*. [6] were the first to study deceptive movement and anticipation in sport, using temporally occluded videos of an attacker approaching a camera, the perspective of a defender. Participants were given the role of the defender and required to predict the side which the attacker would pass, left or right. The attacker would undertake a non-deceptive movement or a deceptive movement. From these findings, it was concluded that expertise may affect anticipation ability, where experts more accurately detect final passing direction when compared to novices [6]. Since, the study of perception, anticipation and deceptive motion within sporting contexts has been further studied, confirming the superiority of expertise [9, 15–18]. However, few studies have considered the underlying mechanisms to explain these findings [8, 10, 19, 20]. Huys *et al*. [19] studied the manipulation of corresponding dynamics of a tennis serve through point-light displays. They reported that spatial occlusion impacted the dominant dynamics, additionally, suggesting cue information pick up was more global (i.e., absolute body displacement referred to as a whole single object) than local (i.e., body displacement with additional cue information from limb joint articulations). Furthermore, Lopes *et al*. [20] performed a biomechanical analysis of penalty kicks in soccer and reported that limbs providing local relative information were modified during the early stages of approach for deceptive motion. Finally, Brault *et al*. [8] reported that the performance of experts correlated with the evolution of the perceptual variable tau (cf., [21]), that is, experts fixated on honest signals from global motion (e.g., center of Gravity (CG)) than local ones (e.g., limbs). To detail, global motion cues refers to absolute whole body movement and local motion cues refer to information pick up from body parts or regions [19]. While previous studies have studied the possible differences of global and local motion cues, and their potential links to expert performance, no studies have investigated the role of direct access to global motion cues such as CG or their possible training effect in competitive settings.

In non-competitive settings, previous studies have considered the direct access and influence of global motion cues on behaviour and action responses. Savelsbergh *et al*. [22] showed that subjects could regulate their hand aperture through optical size expansion alone (i.e., tau [21]). Additionally, Lynch *et al*. [23] studied interactions of a crossing task with varying global and local obstacle appearances, a cylinder, CG, a virtual human, the trunk alone and the legs alone, within virtual reality. They reported differences of crossing distance between the global and local visual cue appearances but reported no difference in the ability to complete the task. Finally, Meerhoff *et al*. [24] also studied the role of global and local motion information during a distance regulatory locomotor task. Their findings suggested that ability to complete the task, while interacting with a large sphere or a virtual human, was not impeded; however, there was a temporal advantage when both local and global motion cues were available. Training through virtual reality allows for additional enrichment of the environment (augmentation), an ability to reduce the task to several smaller steps (simplification) and change the speed or distort the environment (variability) [25]. Additionally, virtual reality allows for judgements to be assessed through simulated action responses, although this may lead to biased results [26], better anticipation skills remains widely debated within the literature, through visual expertise or motor action capability expertise [27].

### Objectives and contribution

From the work of [8], there was a reported correlation tau and its rate of gap closure of the attacker’s CG with the rate of success in expertise. This observed correlation is interesting as it can be interpreted that global absolute displacement of CG may be perceptually important, a focal point of attention during interaction, which in turn can be treated as a trainable variable to attune defender attention. Therefore, the objectives of this study are first to isolate CG and present an attacker’s absolute global motion in the form of CG only to expert and novice defenders. The isolation and visualisation of CG will allow us to investigate whether CG alone is sufficient to detect deceptive motion. Secondly, previous studies showed that the performance of novices was correlated with the tau of upper trunk motion. Therefore, our second aim is to investigate whether the presence of CG, as an additional visual cue on an attackers body, could act as an aid where we would expect a more significant increase of correct response performance for novices.

To answer these questions, we proposed to manipulate the visual appearance of the attacker. To preserve and repeat kinematic motion while manipulating visual appearance, we conducted our experiment in virtual reality. The application of virtual reality in ecological perception and action has been proven a powerful tool [28] although it is not without limitations, as there exist variances in depth perception [29] and forward gaze-driven speed is underestimated compared to actual speed [30]. Considering these notable variances, virtual reality has been validated to conform with reality; a sense of personal space [31], interactions with virtual humans [23, 32, 33] and within competitive settings [8].

We completed two experiments, both in virtual reality, where participants were presented a dynamic virtual attacker on an approach to a defender (the participant). Between trials, the visual appearance of the virtual attacker would vary the amount of global and local visual cues available. The first experiment was a task that required no movement by the participant, which we have referred to as the perception task. Participants recorded their responses through a peripheral device, this first task aimed to identify variances from the manipulation of available perceptual information. We used this paradigm since our experiment involved both inexperienced novices and highly competitive experts, as specific action responses observed in the perception-action condition could be attributed to the physical (action) capabilities [27]. The second experiment focused on a simulated movement task, which for the paper we refer to as the perception-action task. The position of the participant was tracked during the task, investigating perceptual expertise with response times. This experiment was designed to complement the results of the first experiment and to consider the results of previous works that showed the importance of preserving the representativeness of experimental task designs when exploring the performance of the athletes, particularly in time-constrained actions [34, 35].

In that context, we formulated two hypotheses:

- H1: considering the previous findings of a correlation between CG motion and performance in expert rugby players [8], we hypothesized that performance will be decreased for novices whilst interacting with only a visual representation of CG.
- H2: we hypothesize that the presence of CG will act as an aid and the performance of novices will be greater whilst interacting with a combination of a virtual attacker and CG than whilst interacting with a virtual attacker alone.

Our first contribution is to design an experimental platform in virtual reality to study the influence of global and local motion cues within a rugby attacker-defender dyad task. Our second contribution is that while global motion can be visualized and acted upon, there is insufficient information to maintain success rate. Local cues such as limbs motion and trunk orientation are necessary for effective perception and perception-action tasks. To demonstrate this, we have formulated the paper in the following way: the method section describes both perception and perception-action tasks separately, highlighting their differences. Results from the perception and perception-action task are presented as separate sections, and we discuss the findings collectively in the discussion.

## Method

### Participants

Seven expert rugby players aged 24.71±2.43 (mean±SD) and nine inexperienced subjects aged 27.56±10.69 years participated in this study. All participants were male, had normal or corrected-to-normal vision and no history of disease or impairment which could have affected their ability of participation. The expert rugby players competed regularly in the French second national league (ProD12) with 16.29±4.23 years of experience in playing rugby. Novices were university staff and students with no prior experience of competitive rugby. This study was carried out in accordance with the recommendations of the research institute. All participants gave their written informed consent in accordance with the declaration of Helsinki.

### Apparatus

**Fig 1:**
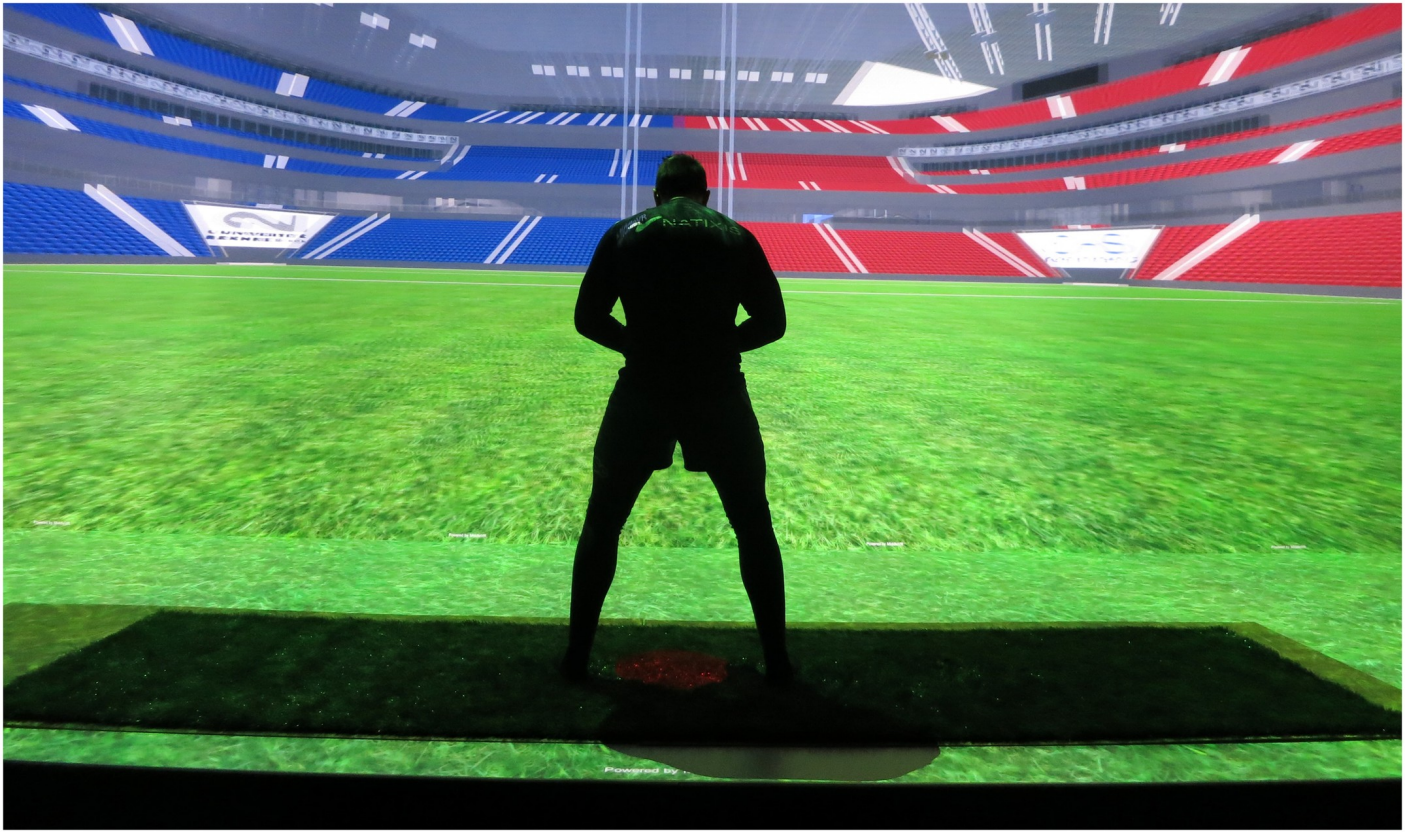
Immersed participant. Third person perspective of a participant immersed within the virtual environment.

Experiments took place in a 4-screen Computer Assisted Virtual Environment (CAVE), which was 9m wide, 3m high and 3m deep. The CAVE was fitted with a synthetic all weather turf surface for increased immersion during experimentation. It was equipped with 13 projectors with 15MPixels resolution in total. The 3D environment display and character animation (Fig 1) was designed in the Unity game engine. Multi-surface rendering was performed by the MiddleVR plugin. Active stereoscopy was achieved with Volfony ActiveEyes Pro Radiofrequency wearable glasses. Glasses were tracked by a 16 camera ART tracking system.

### Stimulus

For all trials, an attacker was positioned in front of the participant and advanced towards the participant after a visual countdown of three seconds. Attacker motion was realized through previously captured motion of eight French national league rugby players [8] using the optoelectronic motion capture ViconMX system (Oxford Metrics, Oxford, UK). A total of 16 motions were presented as stimuli; attackers either performed one of 8 deceptive motions (DM), otherwise known as a side-step, or one of 8 non-deceptive motions (NDM), no change of direction after initial reorientation. Several attacker motions were used, opposed to the repetition of one attacking motion, to reduce possible identification of repeated motions.

### Appearances

**Fig 2:**
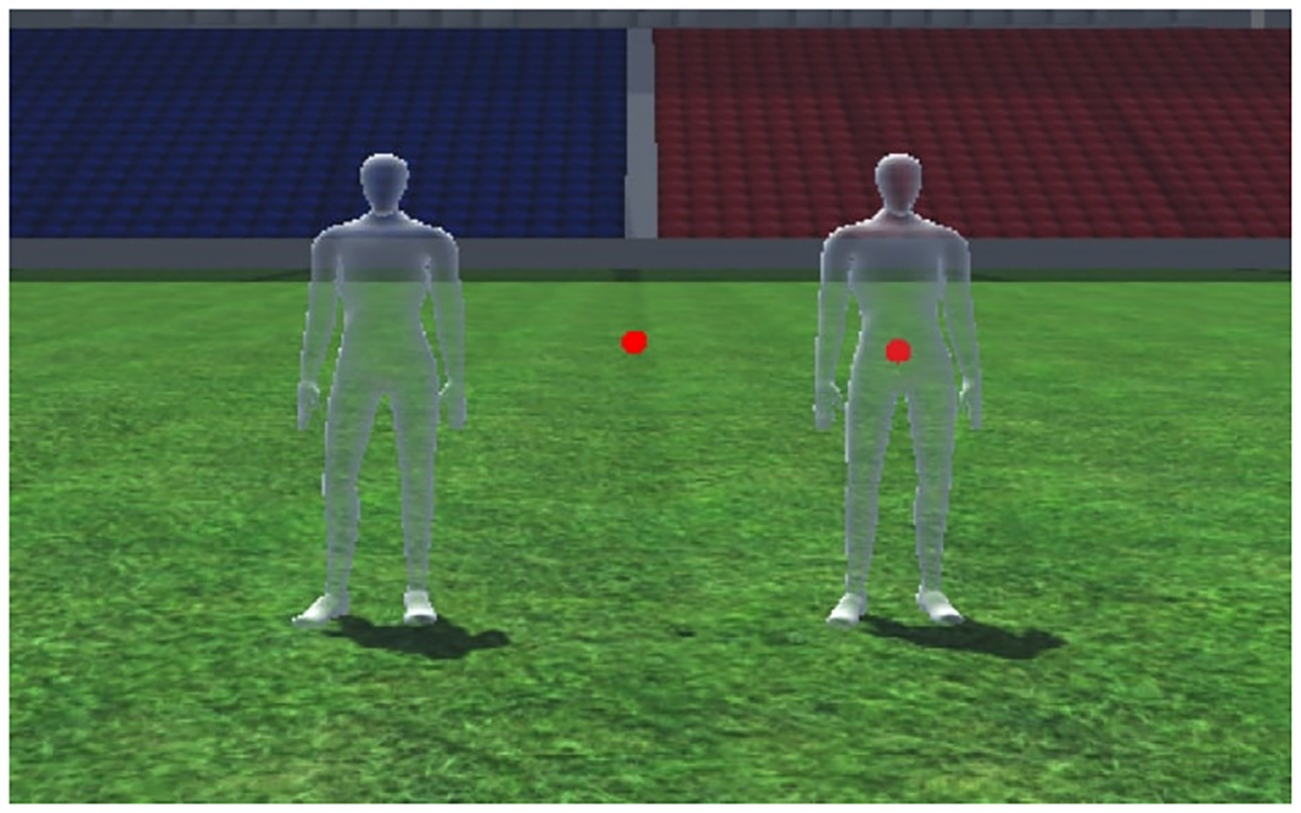
Visual appearances of stimuli. Visual appearances of stimuli: full body (left), center of gravity (center), full body and center of gravity combined (right).

To investigate whether the center of gravity is sufficient for the detection of deceptive motion and if it can be used as a training aid, we have manipulated the visual appearance of a virtual attacker using the following conditions, as illustrated in Fig 2:

1. Full body: A mobile mannequin with a shoulder width of 37cm and hip width of 27cm, was rendered without a face to avoid any influence of gaze direction. Furthermore, the motion was animated as carrying an invisible ball to maximize character perception and reduce occlusion of character motion by the presence of a rugby ball.
2. Sphere: With recent findings of Brault and colleagues [8] suggesting a strong correlation between CG and interception during a deceptive sporting task in terms of success, we chose to remove all body encasing information and use a small sphere (8cm diameter) as an appearance of CG. Such a reduction would remove all information including, orientation and ground contact.
3. Combination: Both a full body attacker with their CG are presented to the participant, a combination of orientation and ground contact with the less deceptive motion of CG being present.

Note that condition 1) served as a control condition, using a virtual human (VH), condition 2) completely removed any local motion cues leaving only global information of motion (CG), finally, condition 3) combined both VH local and CG global motion cues to maximize stimuli (HC).

### Perception task

Participants were immersed in a virtual environment (Fig 1), where they were instructed to stand in a designated location, as indicated by a red circle on the floor of the immersive environment that remained visible throughout. Using a Latin square design, experimentation divided into three randomized blocks of attacker appearance (Fig 2). Within the perception only task for each visual appearance, we also set conditions based on an occlusion time paradigm, allowing to test for the quantity of visual information on decision processing for future actions. The moment of toe off, as the attacker’s foot leaves the ground, before reorientation occurred was used as a reference point for all attacker motions (T0). Thereafter, our three experimental conditions consisted of the duration of visual appearance before occlusion, cut off times were 100ms (T1), 200ms (T2), and 300ms (T3). These cut off times were selected for the full completion of whole body reorientation after T0 [7], and to investigate the effect of appearance on the time needed for decision making. Participants were informed that they would be acting as a defender and would be required to detect the direction which the attacker would go after occlusion for a successful interception. There was no feedback of success or failure throughout the experiment. For each visual appearance, a total of 16 attacking motions (8 DM and 8 NDM) were repeated with three different occlusion cut off times (100ms, 200ms, and 300ms). After occlusion participants were required to indicate, using the hand-held wand, which direction the attacker was going to pass. Participants were given a period of familiarization prior to data capture.

For each block of visual appearance, these conditions were presented randomly within and between subjects. In total, each participant performed 144 trials (3 appearances x 3 cut off times x 16 motions). The duration of the experiment per participant, including training and resting periods, was 30 minutes.

### Perception-action task

Participants were immersed in the same virtual environment, and the appearances were presented in the same order as the perception task, the conditions per block were randomized independently. For the perception-action task, there was no occlusion paradigm, and the attacker would approach and pass the defender. Participants were informed that they maintained the role of defender, however, in this instance the attacker would not disappear but continue running. Participants were then instructed to move medio-laterally in an attempt to intercept the attacker. Participants wore a belt fitted with reflective markers, and their medio-lateral displacement was tracked using the 16 camera ART tracking system. We used the same threshold of displacement velocity as [8], with speeds greater than 0.5m/s were used to record initial response after T0. Additionally, response times were recorded at the instance of the initial response. Similar to the perception task, participants were provided a period of familiarization to adapt to the perception-action task.

Similarly, for each block of visual appearance, these conditions were presented randomly within and between subjects. In total, each participant performed 48 trials (3 appearances x 16 motions). The duration of the experiment per participant, including training and resting periods, was 15 minutes.

### Analysis

Perception responses and perception-action belt motion were post-processed using customized MATLAB scripts (Mathworks 2016b), responses were then expressed as a percentage of success (mean±SD). We set the level of significance to *α* = 0.05, Bonferroni corrections were used to reduce type I errors, with the adjusted *α* reported for main effects and interactions in the results [36]. Further, a Bonferroni correction was applied for post-hoc tests. A Shapiro-Wilk test was performed to evaluate whether data followed a normal distribution. For both experiments, the dependent variable was the rate of success, and a general linear mixed model analysis was used to determine the effect of appearance, type of motion (DM and NDM) and cut off times for the perception task. For the perception-action task, rate of success and response time were the dependent variables to determine the effect of appearance and motion. Mauchly’s test of sphericity was used for assumption, and a Greenhouse-Geisser was used as a correction when sphericity had been violated. Finally, effect sizes were computed as partial eta squared $\eta_{p}^{2}$ and pairwise comparisons were used for further post-hoc analysis.

## Perception results

Overall, there was a main effect of expertise (F(1,14) = 31.119, p<0.001, $\eta_{p}^{2}=0.69$), appearance (F(2,28) = 127.826, p<0.001, $\eta_{p}^{2}=0.90$, *α* = 0.004), cut off times (F(1.232,17.247) = 33.115, p<0.001, $\eta_{p}^{2}=0.70$, *α* = 0.004) and motion (F(1,14) = 541.149, p<0.001, $\eta_{p}^{2}=0.98$, *α* = 0.004). Overall, experts were more successful (75.30±31.92%) than novices (61.27±38.13%)(p<0.001), appearance CG had a smaller success rate (48.57±35.72%) than VH (76.43±31.67%) and HC (77.21±33.69%)(p<0.001), cut off time T3 had the greatest rate of success (75.65±35.83%), than T2 (68.75±35.31%)(p<0.001) and T1 had the lowest rate of success (57.81±35.46%)(p<0.001), and non-deceptive motion was greater (92.27±14.41%) than the deceptive motion (42.53±34.23%)(p<0.001). There was no four-way interaction between all dependent variables (S1); visual appearance, cuts off times, motion type and expertise level (F(4,56) = 0.391, p = 0.814).

**Fig 3:**
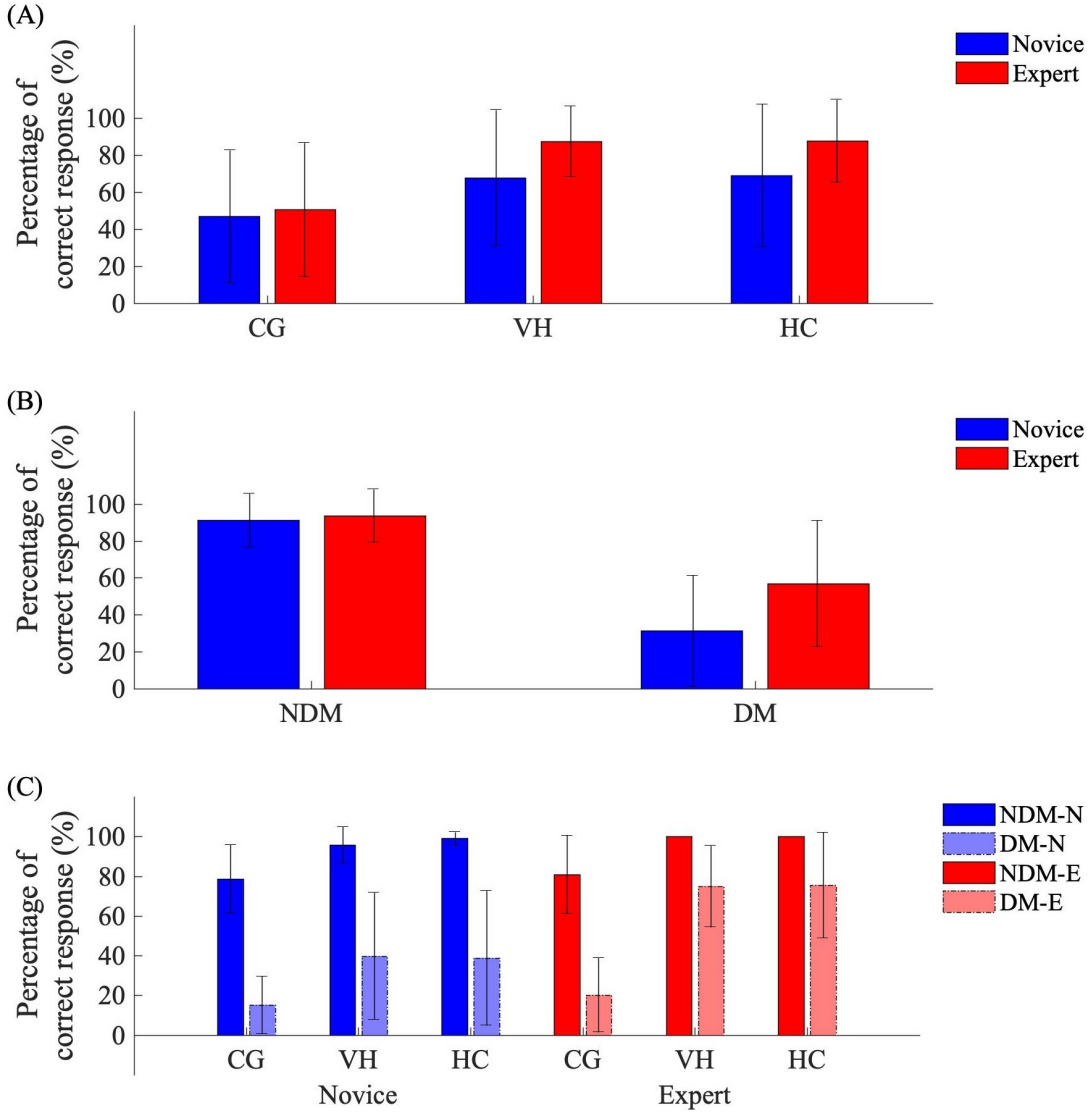
Effect of expertise on rate of success during the perception task. Mean±SD percentage of success when considering effect of expertise during the perception task, for interactions between level (novices (N) blue and experts (E) red) and appearance (A), motion (DM and NDM) (B), and the interaction between level, appearance and motion (C).

Further investigating the main effect of expertise, there was a significant two-way interaction between the visual appearance and the expertise (Fig 3A) of the participant (F(2,28) = 9.166, p<0.001, $\eta_{p}^{2}=0.40$, *α* = 0.007); where we observe similar rates of success for experts (50.60±36.10%) and novices (46.99±35.68%) whilst interacting with CG, and experts were more successful than novices whilst interacting with VH (87.50±19.13% and 67.82±36.63%, respectively) and HC (87.80±22.34% and 68.98±38.60%, respectively).

There was a significant two-way interaction between motion (Fig 3B) and expertise (F(1,14) = 31.144, p<0.001, $\eta_{p}^{2}=0.69$, *α* = 0.005); where we observe similar rates of success for experts and novices during non-deceptive motion (93.65±14.35% and 91.20±14.45%, respectively), while experts were more successful than novices for deceptive motion (56.94±34.11% and 31.33±30.05%, respectively). However, there was no significant two-way interaction between cut off times and expertise (F(1.232,17.247) = 2.710, p = 0.112).

When considering the three-way interaction between expertise, appearance and motion (Fig 3C), there was a significance (F(1.443,28) = 7.795, p = 0.006, $\eta_{p}^{2}=0.36$, *α* = 0.008); where we observe that experts and novices had an increased rate of success when interacting with VH (100% and 95.83±9.17%, respectively) and HC (100% and 99.07±3.34%, respectively) than CG (80.95±19.61% and 78.70±17.27%, respectively) during non-deceptive motion, however, experts had a greater rate of success from interacting with CG (20.24±18.74%) to VH (75±20.54%) and HC (75.60±26.66%) during deceptive motion than novices (15.28±14.43%, 39.81±31.97% and 38.89±33.85%, respectively).

Irrespective of expertise, there was a significant two-way interaction between the visual appearance and cut off times (F(4,56) = 9.971, p<0.001, $\eta_{p}^{2}=0.42$, *α* = 0.005); where we observe that the rates of success were fewer whilst interacting with CG for T1 (46.88±18.78%), T2 (46.48±39.17%) and T3 (52.34±44.73%) than VH and HC, which had similar rates of success for T1 (62.50±39.78% and 64.06±41.61%, respectively), T2 (78.52±25.25% and 81.25±29.61%, respectively) and T3 (88.28±22.44% and 86.33±24.25%, respectively).

Additionally, there was a significant two-way interaction between the visual appearance and motion (F(1.443,28) = 13.969, p<0.001, $\eta_{p}^{2}=0.50$, *α* = 0.006); where we observe that CG during non-deceptive motion (79.69±18.16%) had a lower rate of success than VH (97.66±7.13%) and HC (99.48±2.52%), during deceptive motion this difference became greater between CG and the VH and HC counterparts (17.45±16.46%, 55.21±32.50% and 54.95±35.71%, respectively).

**Fig 4:**
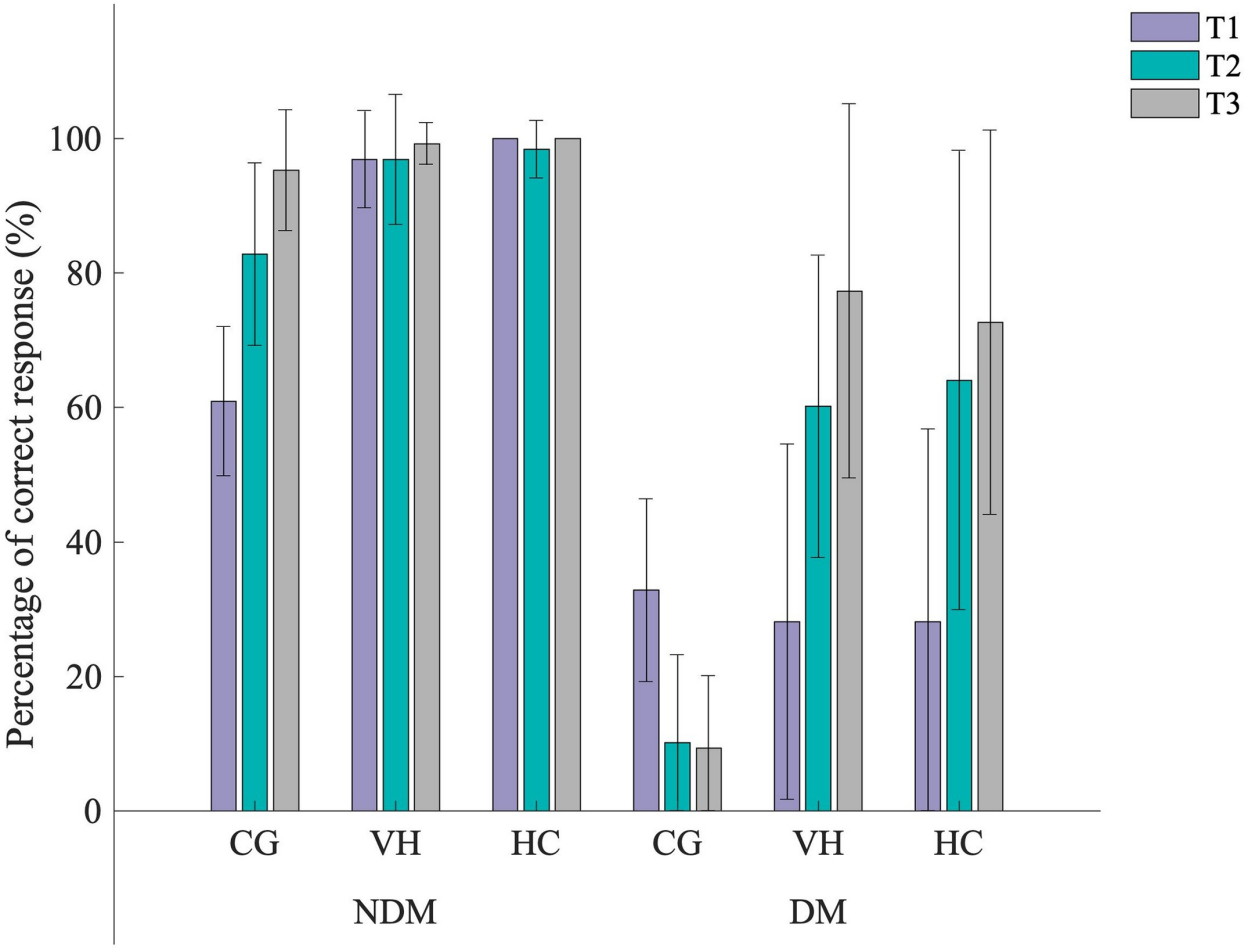
Interaction between appearance, motion and cut off times during the perception task. Mean±SD percentage rate of success for the interaction between appearance, motion and cut off times during the perception task.

Developing from the two-way interactions, there was an interaction between appearance, cut off times and motion (F(4,56) = 41.932, p<0.001, $\eta_{p}^{2}=0.75$, *α* = 0.006)(Fig 4); where, during non-deceptive motion the rate of success was similar for VH and HC at T1 (96.88±7.22% and 100%, respectively), T2 (96.88±9.68% and 98.44±4.27%, respectively) and T3 (99.22±3.13% and 100%, respectively), where CG increased cut off times (60.94±11.06%, 82.81±13.60% and 95.31±8.98%, respectively). During deceptive motion, at T1, CG, VH and HC were similar (32.81±13.60%, 28.13±26.42% and 28.13±28.69%, respectively), at T2, CG had a smaller success rate (10.16±13.09%) than VH (60.16±22.46%) and HC (64.06±34.12%) and at T3, CG had a smaller success rate (9.38±10.70%) than VH (77.34±27.85%) and HC (72.66±28.58%). The rate of success during non-deceptive motion for all appearances and cut off times was greater than those observed during deceptive motion.

## Perception-action results

### Performance

There was no three-way interaction between expertise (S2), visual appearance and motion (F(2,28) = 1.131, p = 0.337). Overall, there was a main effect of expertise (F(1,14) = 9.744, p = 0.008, $\eta_{p}^{2}=0.41$), experts had a greater rate of success (86.01±19.25%) than novices (65.63±32.79%). There was a main effect of motion (F(1,14) = 77.260, p<0.001, $\eta_{p}^{2}=0.85$, *α* = 0.008), non-deceptive motion had a greater rate of success (94.53±10.91%) than deceptive motion (54.69±30.58%). There was no main effect of visual appearance (F(1.427,28) = 4.693, p = 0.031, $\eta_{p}^{2}=0.25$) between CG (70.31±32.03%), VH (78.91±28.12%) and HC (74.61±31.19%).

**Fig 5:**
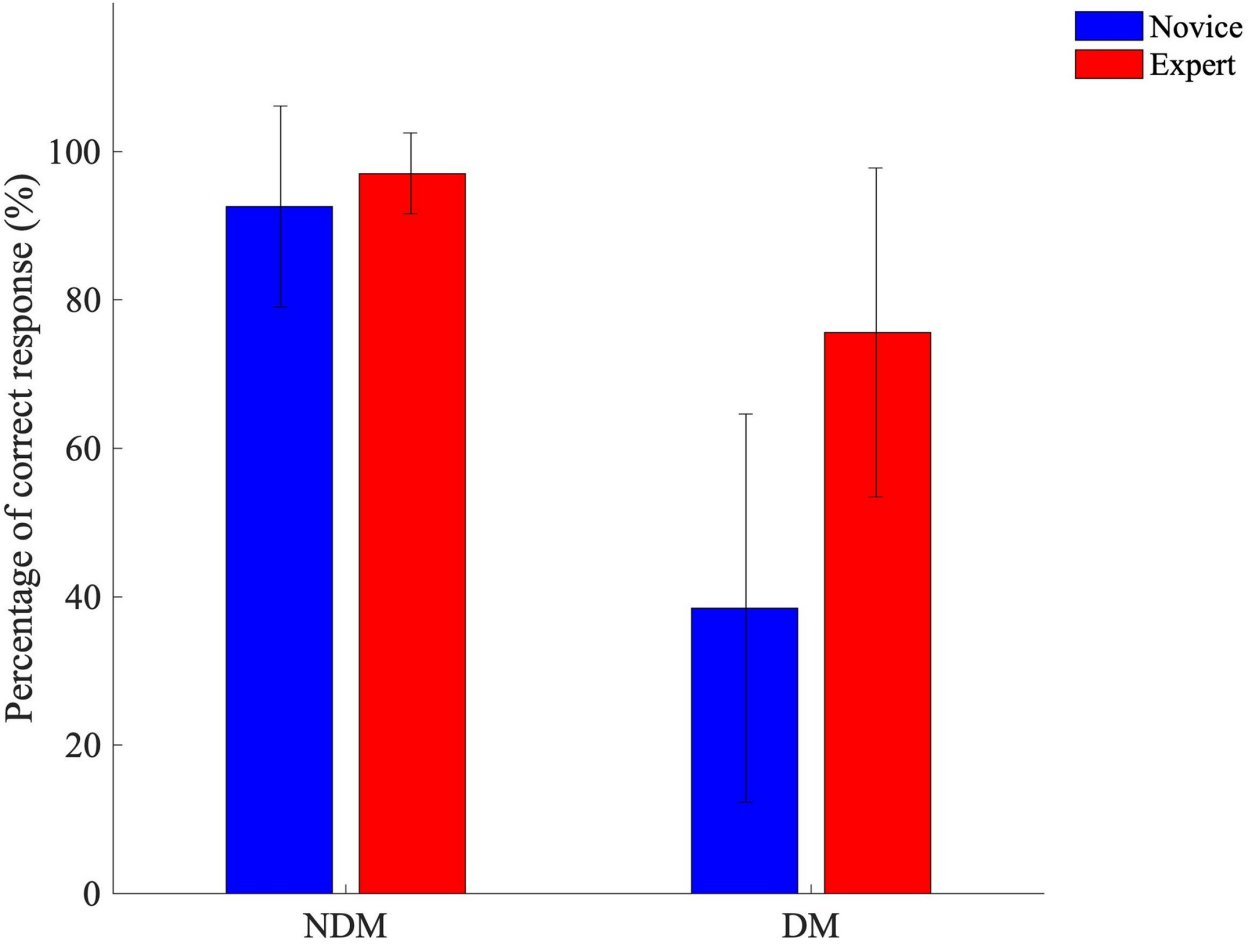
Interaction effects during the perception-action task. Mean±SD percentage of success when considering effects observed for interactions during the perception-action task between level and motion.

There was a significant interaction between motion and expertise (F(1,14) = 14.490, p = 0.002, $\eta_{p}^{2}=0.51$, *α* = 0.010)(Fig 5), where we observe that experts and novices had greater rates of success during non-deceptive motion (97.02±5.46% and 92.59±13.54%, respectively) than during deceptive motion where experts had a greater rate of success (75.60±22.18%) than novices (38.43±26.16%). Considering two-way interactions, there was no significant interaction between visual appearance and expertise (F(1.427,28) = 1.320, p = 0.280). Further, there was no significant interaction between visual appearance and motion (F(2,28) = 2.499, p = 0.100).

### Response time

**Fig 6:**
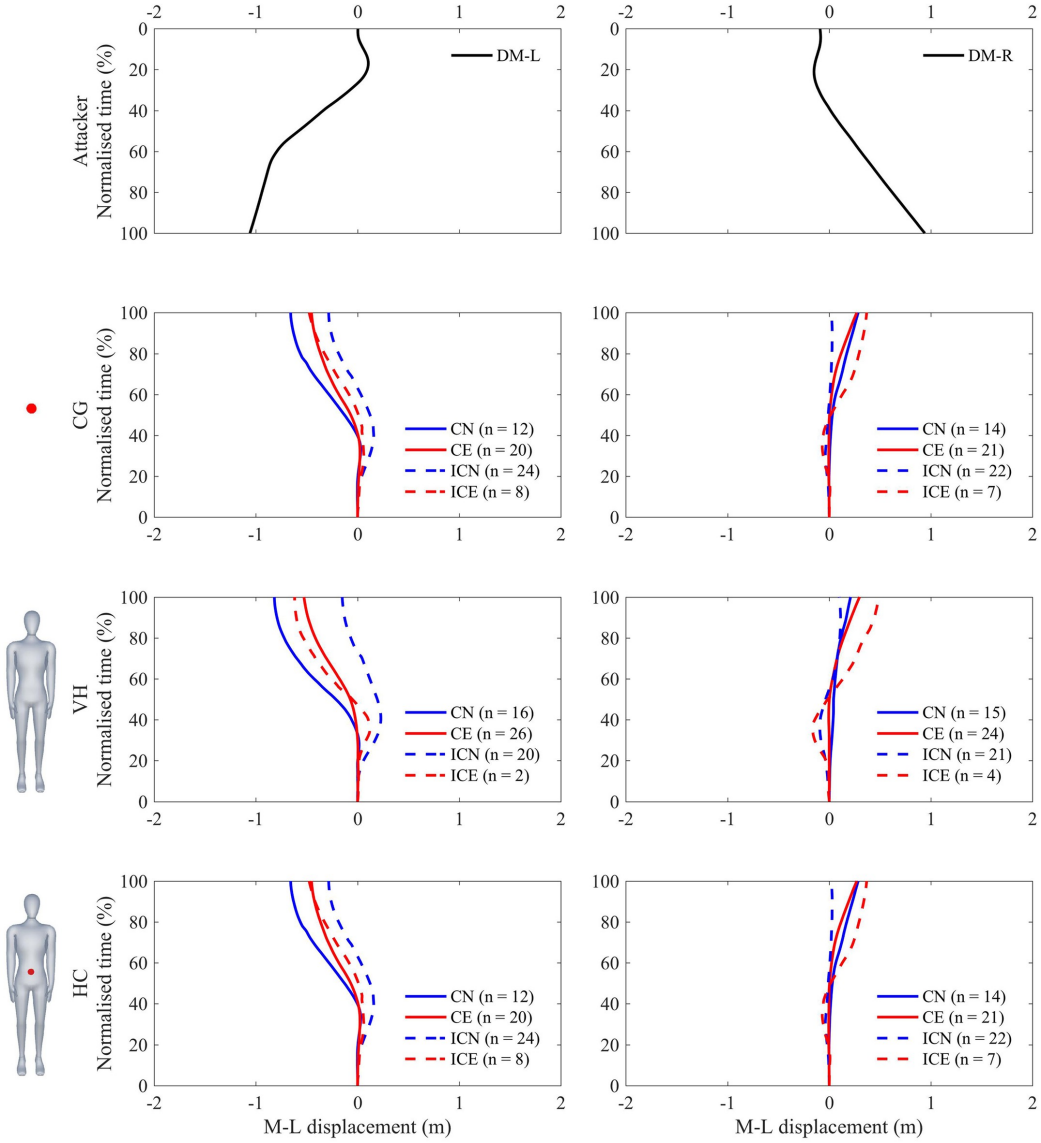
Average medio-lateral response displacement during deceptive motion. Average medio-lateral displacement during normalized time of expert (red) and novice (blue) defenders in successfully (solid line) and unsuccessfully (dashed line) detecting final crossing direction of the attackers motion (top). Columns (left—right) represent the motion of the attacker; DM-L and DM-R, and Rows (top—bottom) represent the visual appearance of the attacker; CG, VH, and HC.

**Fig 7:**
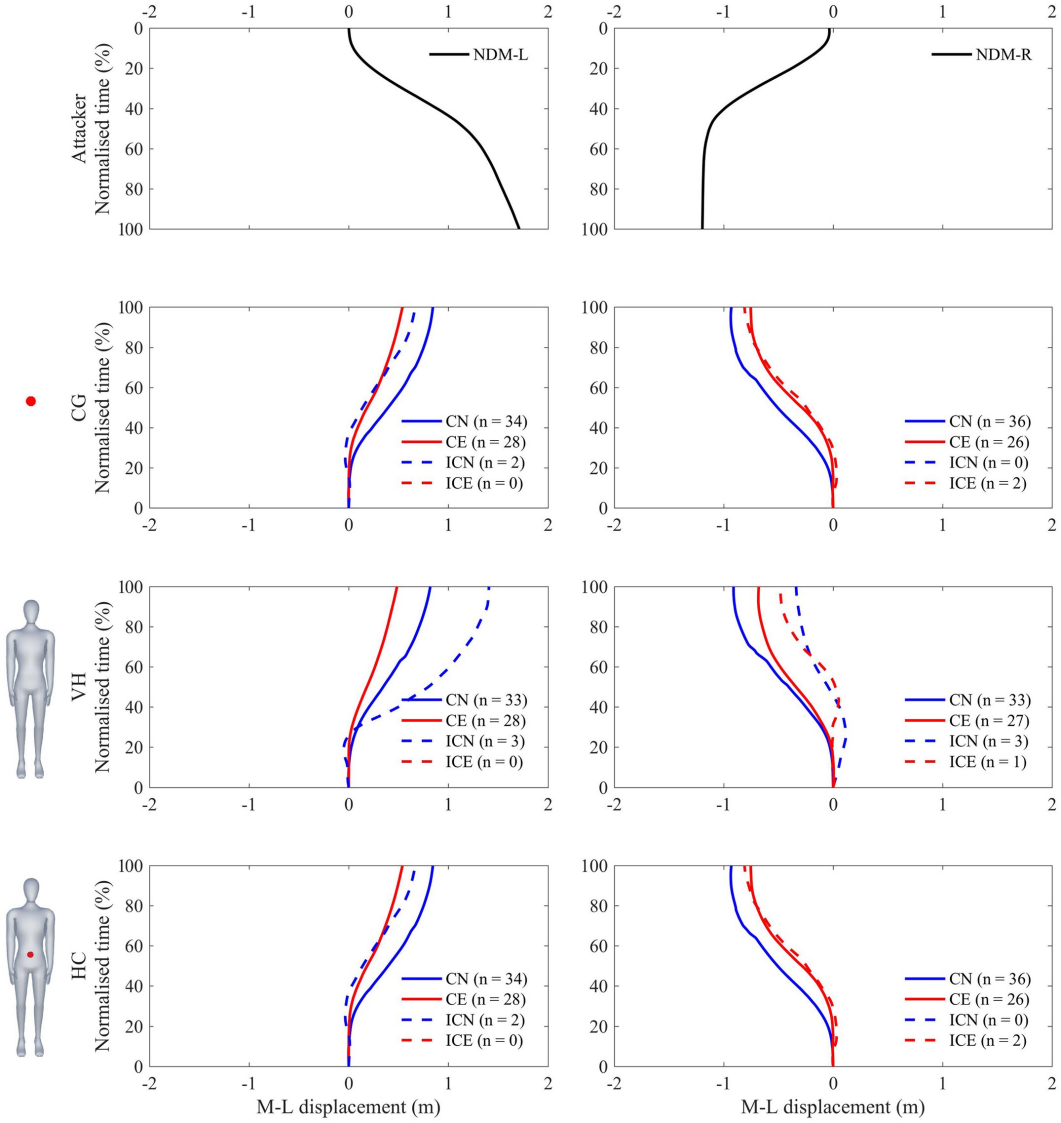
Average medio-lateral response displacement during non-deceptive motion. Average medio-lateral displacement during normalized time of expert (red) and novice (blue) defenders in successfully (solid line) and unsuccessfully (dashed line) detecting final crossing crossing direction of the attackers motion (top). Columns (left—right) represent the motion of the attacker; NDM-L and NDM-R, and Rows (top—bottom) represent the visual appearance of the attacker; CG, VH, and HC.

Figs 6 and 7 presents the mean medio-lateral displacement of the defender as a function of normalized time, showing average correct and incorrect responses for both novices and experts. There was no significant three-way interaction (S3) between expertise, appearance and motion on response times (F(2,28) = 0.405, p = 0.671). Independently, there was no main effect of expertise (F(1,14) = 2.906, p = 0.110). There was an effect of appearance (F(2,28) = 89.224, p<0.001, $\eta_{p}^{2}=0.86$, *α* = 0.013), CG had a later response time (0.71±0.15s) than VH (0.53±0.16s) and HC (0.52±0.14s). Finally, there was a main effect of motion (F(1,14) = 36.839, p<0.001, $\eta_{p}^{2}=0.73$, *α* = 0.008), where deceptive motion had later response times (0.63±0.18s) than non-deceptive motion (0.54±0.15s).

There were no significant two-way interaction between expertise and motion (F(1,14) = 1.204, p = 0.291), expertise and appearance (F(2,28) = 1.540, p = 0.232) or motion and appearance (F(2,28) = 1.537, p = 0.233).

## Discussion

The present study focused on the visual cues conveyed by an attacker within a rugby dyad. We replicated an attacker-defender dyad, similar to [8], through virtual reality. In [8], they implemented such a scenario using head-mounted displays (HMD), they recruited 28 participants for the perception only task (14 experts and 14 novices) and 24 participants for their perception and action task (12 experts and 12 novices), and they concluded that there is a relationship between attacker CG and expert defender rate of success. Developing from this, we focused on the visual representation of an attacker, a general full body, a full body with CG visible or only the CG of the attacker being present. We then evaluated the influence of these visual manipulations under two similar tasks, a perception only task and a perception-action task. For our study we had 16 participants that completed both tasks (seven experts and nine novices). We had hypothesized that CG alone would not impede the performance of experts and we had further hypothesized that the presence of CG with a full body representation would improve the performance of novices. From our results, we observed that actually, CG impeded the performance of experts. Further, we observed the CG did not act as an aid as there were no performance differences between HC and VH conditions for novices. We will discuss these findings and the influences of the other independent variables from our study.

### Expertise performance

Previously, Brault *et al*. [8] reported that experts were more sensitive in attuning their perceptual skills to more *honest* signals, specifying the medio-lateral displacement of the CG, while the novices were more attuned to deceptive, the otherwise *dishonest* signals, referring to upper trunk and shoulder rotation. Additionally, they reported that experts gained a majority of correct responses sooner than novices, suggesting that experts could accurately anticipate the final crossing direction of the attacker with less information. While, during an action response task, experts waited significantly longer than novices before initiating their interception movement. The results of our study showed a delayed response time that was observed for both novices and experts, with experts initiating displacement later than novices. The delayed responses observed by experts has previously been associated to a method of gaining more reliable information about the perceived action [27], this is coherent with our findings, experts waited longer than novices. However, from our results we observed later response times for novices while interacting with CG than VH and HC, where they may well be incorporating a similar method of gaining more reliable information. The observed differences between novices and experts in this study may be further explained by the action capabilities of experts, such as being physically able to cover greater distances in shorter times [37–39].

Within our study, experts were more successful in identifying the future passing direction of an opponent than novices. These findings were consistent for both perception and perception-action tasks. Novices and experts were equally successful in determining the passage of the attacker, irrespective of visual appearance or cut off times during NDM. The difference in expertise only became distinguishable during DM, where performance for both experts and novices significantly decreased, experts were superior to novices in anticipating deceptive motion. These findings are in accordance with previous literature, that experts have a greater rate of success in identifying and correctly acting up deceptive motion within rugby [6–9, 15], cricket [16, 18], handball [14] and tennis [17].

### Appearance performance

Where [8] previously hypothesized a correlation between CG and performance, we have not produced supporting evidence for this. Our findings report a detrimental effect on performance while interacting with CG. First, in the perception only task, experts and novices had similar performance scores while interacting with CG but experts regained their advantage of expertise while interacting with VH and HC when compared to novices. While novices did improve during interactions with VH and HC compared to CG, they did not improve with the aid of HC when compared to VH. Moreover, the detrimental effect from CG was observable during both deceptive and non-deceptive motion, the success rate in performance decreased the longer the stimulus was visible. During the perception-action task, both experts and novices were most successful while interacting with VH. Novices had a similar success rate between VH and HC, while experts performed best with VH.

In non-competitive settings, [24] and [23] both reported advantages of full body representations as opposed to global motion visual cues. Meerhoff *et al*. [24] reported temporal advantages in completing a mimicry task while interacting with local motion cues (i.e., arms and legs) or a large sphere (i.e., global motion cue) the same height as the virtual human. Lynch *et al*. [23] also reported advantages of full body representation with local cues as opposed to interactions with global motion cues only. Where cues were not bound to a subjective preference of visual appearance, rather, the perceived action-opportunities. Specifically, their visual appearances of local motion cues produced similar results while global motion cues varied. Our findings are in accordance with these previous findings, global motion cues alone lack sufficient information for reproducible and repeatable performances as observed for local motion cues. Additionally, there was no advantage with the simultaneous presence of global and local motion cues. In our specific setting, the loss of orientation information during approach may have affected performance.

### Timing

During the perception-action task experts generally took longer to react to the stimuli, CG and deceptive motion required longer response times. Moreover, both novices and experts required more time to observe CG, for both deceptive and non-deceptive motion. Further, during the perception task there was an effect of cut off times, overall performance improved with longer exposure for both experts and novices. This trend of improvement in performance was observed for both VH and HC, however performance with CG did not improve with time. Rather, when considering visual appearance with DM and cut off times the success rate lowered after T1.

Our findings of response time during the perception-action task were similar to those of [8], experts took longer to act on a decision, they attributed these findings to a superiority in expertise, that is, experts minimised the number of motion errors in the wrong direction by delaying their response. This could also explain the delayed response observed for both experts and novices whilst interacting with CG, both groups delayed their responses to minimise errors in the wrong direction. However, this does not fully explain the differences observed between experts and novices if both are adopting similar strategies.

Meerhoff *et al*. [24] also reported a temporal advantage of local motion cues (i.e., arms and legs) during a non-competitive setting; participants whilst interacting with a large sphere, the same height as the virtual human, had delayed responses in producing a mirrored or mimicked action of the visual display. Further, Lynch *et al*. [23] reported similar findings during an orthogonal collision avoidance task. Participants, while interacting with a small red sphere, delayed their action response than during other interactions of global (i.e., a cylinder) or local motion cues (i.e., virtual human). They attributed this delayed response to a later threshold in the optical expansion than larger obstacles and the action-opportunities perceived by the participant.

Although, in a head on approaching task, Savelsbergh *et al*. [22] has previously shown a correlation of time to contact and the rate of expansion, where participants adjusted their hand apertures to catch a luminous ball. Considering these findings, we suggest that indeed both experts and novices adopted a delayed response to minimize errors whilst interacting with CG. This condition had indeed limited visual information with a low rate of expansion, creating uncertainty about the future passing direction of the attacker. This could possibly explain the inverted performance observed in the perception study where success rate dropped as cut off time increased during CG.

Finally, the observed differences between novices and experts could be explained by experience based on perceived action—opportunities [40–42]. That is, experts contain the appropriate motor repertoire from previous experience to associate from perception to required action. In part, this could explain the observed difference between perception and perception-action tasks; experts were unaccustomed in perceiving global motion of CG alone, however, during perception-action, they access their motor repertoire when physically required to intercept CG. Indeed, interacting with CG would not be habitual for both experts and novices; however, experts are familiar with the attacker-defender task and their action capabilities.

### Limitations

An initial limitation in direct comparison to the previous study of [8] would be the change of apparatus. Previously, [8] completed their virtual reality study through the application of head mounted displays, while we have conducted our study using a computer assisted virtual environment. Both approaches have been previously validated and applied in ecological perception studies [8, 28], with known limitations [29, 30]. Head mounted displays have previously been reported to have a reduced field of view and an increased impact on inducing simulator sickness compared to immersive environments [43, 44]. A second limitation of the study could be attributed to the response times. Within our experimental setup we recorded response times from the moment of toe off before attacking direction reorientation defined as T0. Theoretically, as the stimulus was present prior to T0, participants could have performed an initial movement before an actual change in direction. However, we only considered response times after T0 as the stimulus prior the reorientation provided no additional information on passing direction, this is in accordance with the paper of [8], but it could be interesting to evaluate if response times appeared before T0 for some players, mainly novices. Finally, we observed differences in overall response times in comparison to [8]. We attributed this general difference of mean response times to possible variances in the distance at which T0 occurs, while we maintained a similar methodology in the determination of T0 as [8], there was no specification of distance of the attacker from the defender at T0.

## Conclusion

To our knowledge, this is a first paper to investigate the effect of visualized CG and performance in a rugby attacker-defender dyad. The findings from our control condition were in agreement with previous literature, experts more accurately detected the side an attacker would pass than novices, and that performance is hindered by deceptive motion such as a side-step in rugby. However, the centre of gravity as a global motion cue was not sufficient in correctly detecting attacker direction. The rate of success was least for both experts and novices during deceptive motion whilst interacting with CG. The inclusion of CG on VH (HC condition) had no additional effect on performance or response times for both novices or experts in comparison with VH alone, the decrease in performance could suggest that CG distracted the participants rather than aided them. During the perception-action task both novices and experts adopted a delayed response whilst interacting with CG. Additionally, experts had delayed responses compared to novices for all visual appearances. We attribute this difference to sport specific action-opportunities, experts being trained and adapted to the attacker-defender dyad and may access and use an associated motor repertoire to delay their responses for increased certainty prior to initiation of motion. In comparison to [8] it appears that the global displacement of the whole body is necessary, however, the introduction of CG combined with VH (HC), was inhibitory on overall performance for experts. Our current results suggest that the introduction of CG as a training aid in attacker-defender dyad is not a relevant cue. A long-term training program with the continual application of HC may decrease the observed distraction effect we have reported here. Future work could consider the use of eye tracking as a method of detailing the notions of global and local cues, alternatively, the introduction of rigid attackers, without articulations of the joints, may provide further insight. Where we have been unable to identify a possible training aid through the visual representation of CG, global displacement of a whole body, without articulation, may be a simplification of the interaction and a greater impact on training applications.

## Supporting information

S1 Fig Perception task four-way interaction results on rate of success. (TIF)

S2 Fig Perception-action task three-way interaction results on rate of success. (TIF)

S3 Fig Perception-action task three-way interaction results on response time. (TIF)
