# Supplementary figures and images for: Detection of deceptive motions in rugby from visual motion cues
Source: PLoS One. 2019 Sep 13;14(9):e0220878. doi: 10.1371/journal.pone.0220878 (PMC6743770; doi:10.1371/journal.pone.0220878)

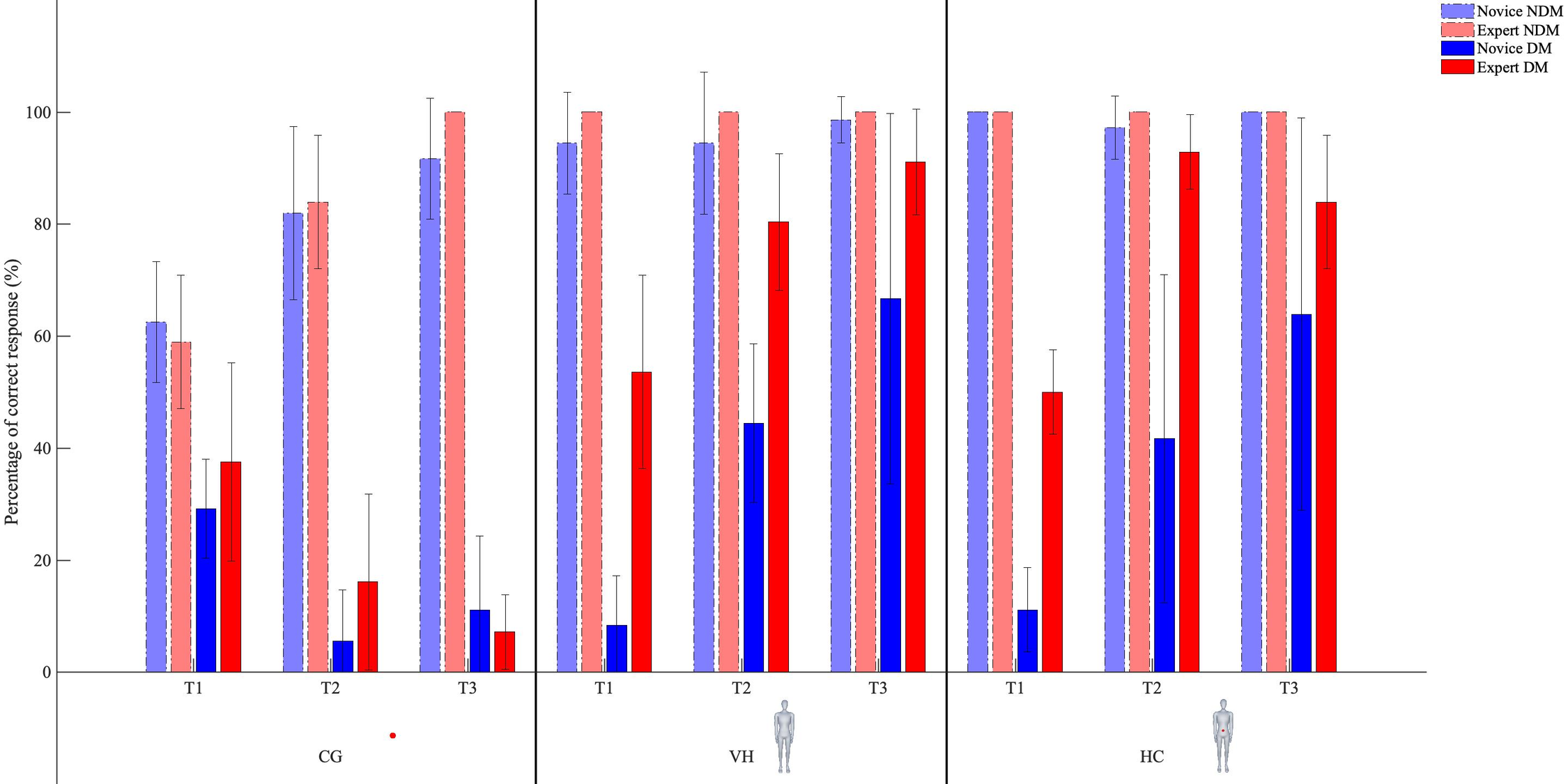

Supplement: S1 Fig — (TIF) [file pone.0220878.s001.tif]

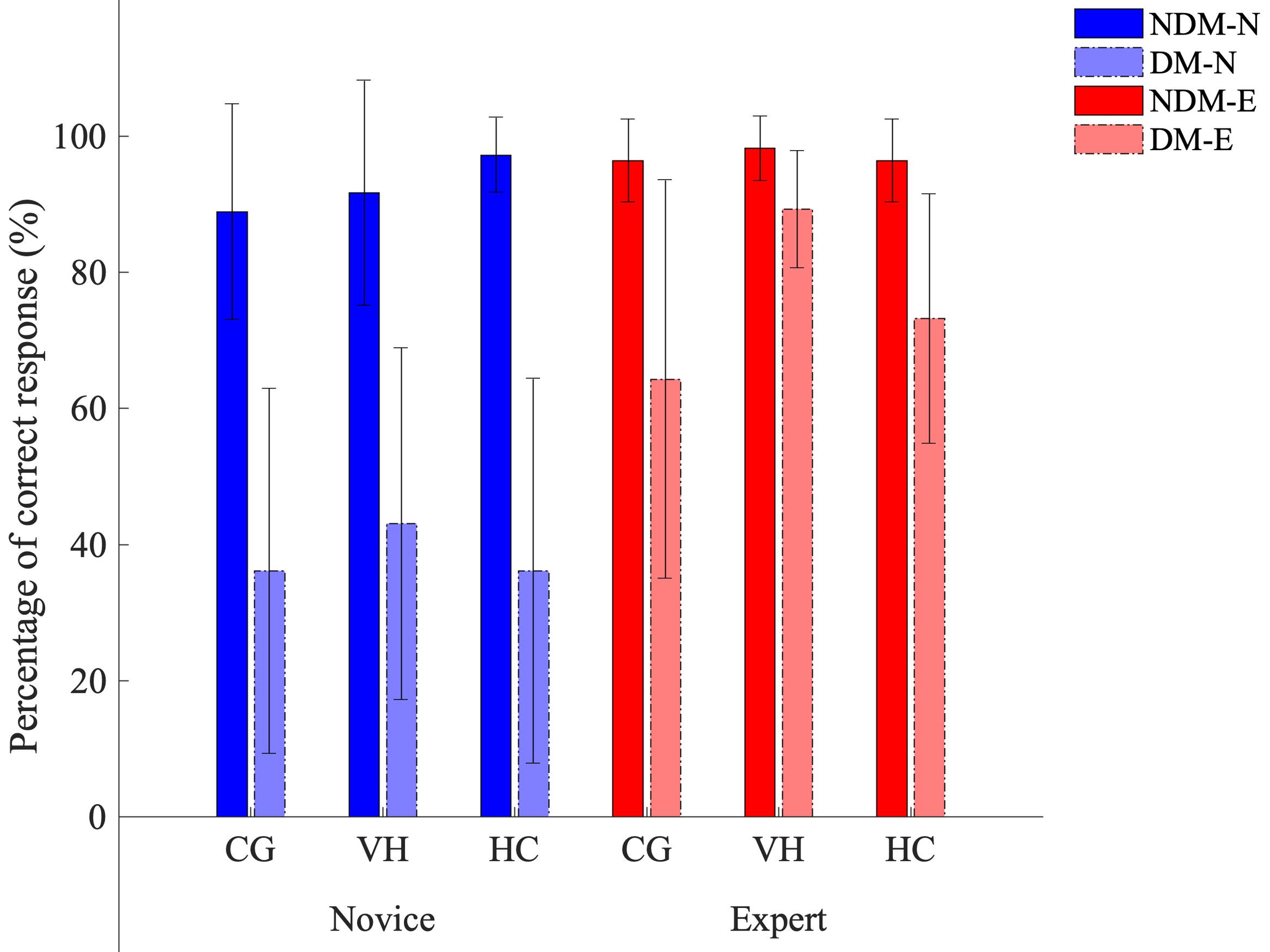

Supplement: S2 Fig — (TIF) [file pone.0220878.s002.tif]

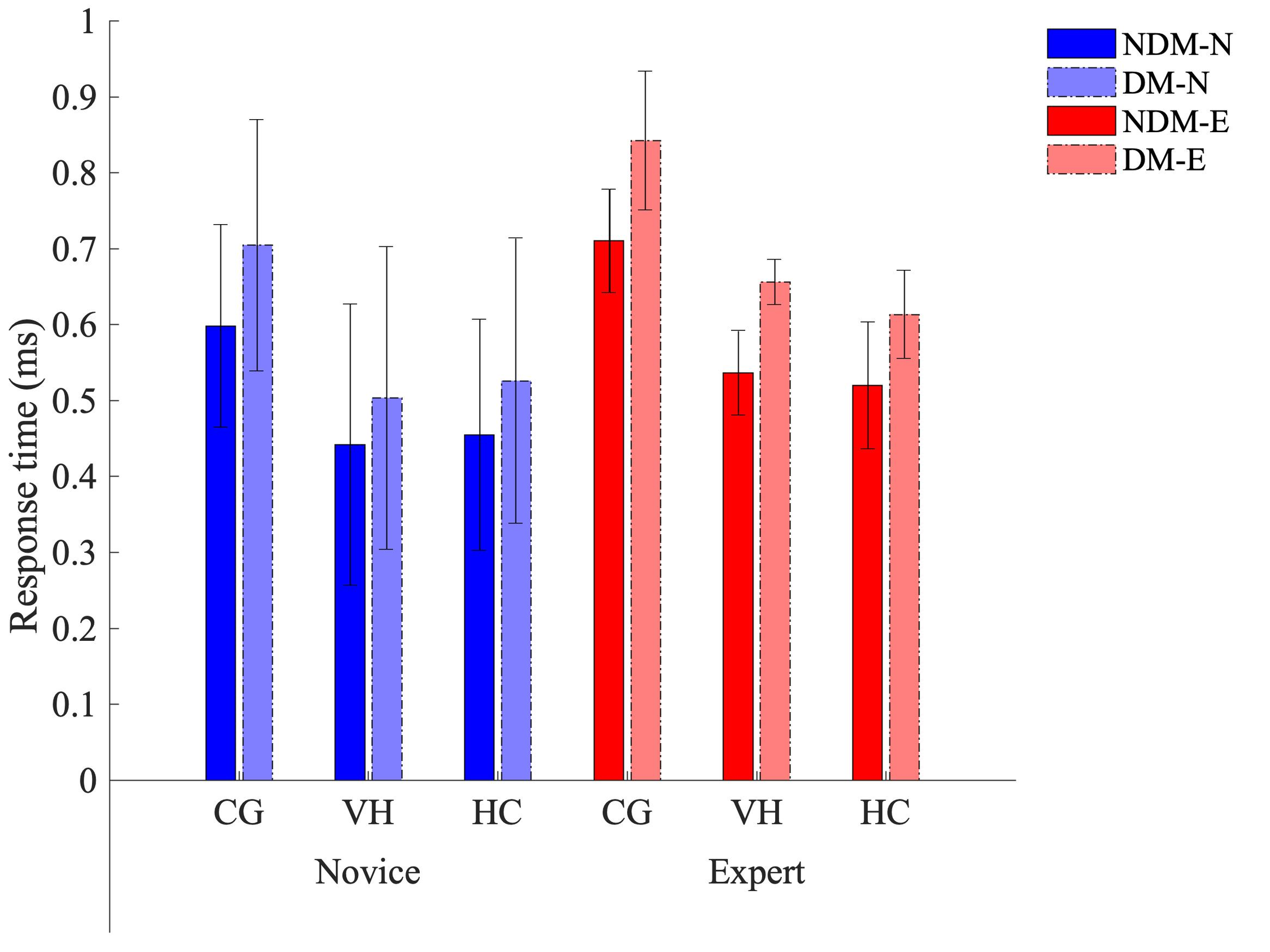

Supplement: S3 Fig — (TIF) [file pone.0220878.s003.tif]
